# BRCA cascade counselling and testing in Italy: current position and future directions

**DOI:** 10.1186/s12885-025-14419-y

**Published:** 2025-07-01

**Authors:** Silvia Costanzo, Lea Godino, Simona De Summa, Tommaso Maria Marvulli, Maurizio Genuardi, Maria Luisa Di Pietro, Emanuela Lucci Cordisco, Daniela Turchetti, Sara Miccoli, Giuseppe Deledda, Giovanna Fantoni, Valeria Viassolo, Oronzo Brunetti, Raffaele De Luca, Maria Digennaro, Francesca Romito, Maria Campanella, Margherita Patruno

**Affiliations:** 1 Heredo-Familiar Cancer Clinic, IRCCS - Istituto Tumori’’Giovanni Paolo II’’, Bari, Italy; 2 Medical Genetics Unit, IRCCS Azienda Ospedaliero-Universitaria Di Bologna, Bologna, Italy; 3 Molecular Diagnostics and Pharmacogenetics Unit, IRCCS - Istituto Tumori’’Giovanni Paolo II’’, Bari, Italy; 4 Department of Life Sciences and Public Health, Università Cattolica del Sacro Cuore, Rome, Italy; 5 Medical Genetics Unit, Department of Laboratory and Hematological Sciences, Fondazione Policlinico Universitario A. Gemelli IRCCS, Rome, Italy; 6 Department of Medical and Surgical Sciences (DIMEC), University of Bologna, Bologna, Italy; 7 Clinical Psychology Unit, IRCCS Sacro Cuore Don Calabria Hospital, Negrar Di Valpolicella, PsyDVerona, Italy; 8 Medical Genetics, IRCCS Sacro Cuore Don Calabria Hospital, Negrar Di Valpolicella, Verona, Italy; 9 S.S.D. C.O.R.O. Bed Management Presa in Carico, TDM, IRCCS Istituto Tumori’’Giovanni Paolo II’’, Bari, Italy; 10 Department of Surgical Oncology, IRCCS Istituto Tumori “Giovanni Paolo II”, Bari, Italy; 11 Servizio Di Psiconcologia, IRCCS Istituto Tumori’Giovanni Paolo II’, Bari, Italy; 12 ETS- Italian National Advocacy On BRCA Carriers. Settimo Milanese-Milano, Settimo Milanese, Italy

**Keywords:** BRCA1/2, Cascade genetic counselling and testing, Communication, Mainstream cancer genetics, Psychological distress, Traditional genetic counselling

## Abstract

**Background:**

Genetic testing has led to a considerable enhancement in the ability to identify individuals at risk of Hereditary Breast and Ovarian Cancer syndrome related to *BRCA1/2* pathogenic variants, thus necessitating personalised prevention programs. However, barriers related to intrafamilial communication, privacy regulations, and genetic information dissemination hinder preventive care, particularly in Italy, where legal constraints limit the disclosure of genetic risks to at-risk relatives. This study examines the relationship between BRCA1/2 carriers’ communication challenges and three factors: cancer status, comprehension of genetic information, and the genetic counseling pathway accessed (Traditional Genetic Counseling, TGC vs. Mainstream Cancer Genetics, MCG).

**Methods:**

This multicenter, prospective, observational study included 277 BRCA1/2 carriers (probands and relatives) aged 18–80 from various Italian centers. Participants completed a sociodemographic form, a self-administered survey, and psychological assessments (Impact of Event Scale, IES and Distress Thermometer, DT). Categorical variables were compared using Pearson’s Chi-squared test or Fisher’s exact test based on sample size and expected frequencies, whereas continuous variables were analyzed using the Wilcoxon rank-sum test because of non-normal data distribution.

**Results:**

Among the 277 carriers (115 probands, 162 relatives), 79.4% received TGC and 20.6% MCG. The cancer prevalence was higher in probands (83%) than in relatives (22%). The probands exhibited greater psychological distress (higher IES and DT scores), and cancer-affected relatives had higher distress levels than healthy relatives (p = 0.008). While no severe psychological distress or PTSD was found, distress was more associated with cancer diagnosis than genetic status. Genetic comprehension was significantly higher in relatives (p = 0.007) and in those who underwent TGC compared to MCG (p < 0.001). TGC carriers also better understood genetic risks and management strategies (p < 0.001).

**Conclusions:**

Psychological distress and genetic comprehension significantly influenced the communication. TGC enhances understanding more effectively than MCG, highlighting the need for tailored support for both carriers and healthcare professionals to improve cascade counseling and testing rates, and cancer prevention. As we look into the future, we need to critically approach MCG, and determine how to address carriers understanding and prevention needs and reincorporate a more comprehensive genetic risk assessment into the MCG model.

**Supplementary Information:**

The online version contains supplementary material available at 10.1186/s12885-025-14419-y.

## Background

Genetic testing has significantly advanced the ability to identify individuals at risk for various diseases such as Hereditary Breast and Ovarian Cancer (HBOC) syndrome. Germline pathogenic or likely pathogenic variants of BRCA1 and BRCA2 (BRCA1/2) are associated with HBOC syndrome, a highly penetrant condition. This syndrome is characterized by a high risk of breast and ovarian cancers in women [1–3], moderately increased risk of breast and prostate cancer in males as well and moderate risk of pancreatic cancer in both genders [4–6]. Therefore, it is imperative for BRCA1/2 carriers to adhere to personalized preventive programs. As HBOC syndrome follows an autosomal dominant inheritance trait, first-degree relatives of BRCA1/2 carriers have a 50% chance of sharing a familial pathogenic variant (PV) irrespective of sex [7].

These advancements highlight the importance of assessing the familial implications when PV is detected. Awareness of carrier status enables informed choices of preventive and reproductive options [8]. Cascade genetic counselling and testing are crucial processes for informing at-risk relatives and enabling them to take preventive or reproductive measures [9]. Specifically, cascade counselling helps inform at-risk individuals and supports them in making informed decisions. Despite these benefits, fewer than half of eligible relatives engage in cascade genetic counselling and testing, largely due to complex family communication dynamics, resulting in missed opportunities for preventive care [10–15]. Consequently, the effectiveness of the proband-mediated approach, in which the first family member tested (proband) is responsible for informing relatives, has been reassessed [10].

BRCA1/2 carriers have traditionally been identified through a complex clinical process known as traditional genetic Counselling (TGC). This process typically involves at least two consultations with a genetics clinician or counsellor. During the pre-test session, the subject's personal and family history was meticulously reconstructed to determine the suitability of recommending a genetic test in accordance with national and international guidelines [16]. For those who underwent genetic testing, a post-test session was organized to disclose test results and discuss their implications for cancer risk management in the counselee and family [17].

However, the advantages of genomic knowledge of personalized medicine render the TGC model incapable of meeting the ever-increasing demands of contemporary cancer genetic practice. To address these challenges, Mainstream Cancer Genetics (MCG) models have been developed [18, 19]. This model enables medical oncologists and other specialists to initiate genetic testing directly on the behalf of patients. In this streamlined approach, the clinician offers a brief pre-test counselling session to explain the purpose of genetic testing. These dual pathways, TGC conducted by geneticists or genetic counsellors and the MCG model facilitated by other specialists, represent complementary strategies that expand access to genetic testing while addressing the complexity of TGC models.

Regarding the psychosocial impact of HBOC syndrome, some studies have shown inadequate rates of familial communication and cascade genetic counselling and testing [20–22]. Conversely, other studies have identified factors that increase familial communication, such as better knowledge of genetics, higher satisfaction with the decision to undergo BRCA1/2 testing, and lower genetic worry [23, 24]. Furthermore, while cancer-affected BRCA1/2 carriers experience psychological distress and burden [25], all BRCA1/2 carriers may feel overwhelmed [26, 27] by the possibility of passing PV on to their offspring. This may cause significant uncertainty regarding risk management [28, 29] and difficulties in family communication regarding the genetic testing results [30–33].

Evidence from the literature [34, 12, 35–38] shows that BRCA1/2 carriers’ major challenges are related to how to communicate genetic information to their family members. Consequently, relatives often receive missing, incomplete, or distorted information regarding their cancer risk. Nonetheless, the moral obligation and responsibility to communicate a genetic diagnosis to family members lies entirely with the carrier himself.

In Italy, privacy regulations and legislation add complexity to the process. By law, genetic information can only be disclosed by the proband, which frequently results in unshared risks within families and missed opportunities for preventive interventions [39]. Italian legislation pertaining to cascade genetic counselling and testing addresses the issues of informed consent to healthcare, the right not to know, and the protection of privacy. Law 219 of the Informed Consent and Advance Directives (2017) mandates that any medical intervention, including genetic testing, can proceed with individuals’ free and informed consent. The law also asserts that every individual possesses the right to be fully informed about health status, including their diagnosis, prognosis, and potential risks and benefits associated with any recommended treatment. Furthermore, the right not to know is also recognized within the aforementioned legislation.

The legal framework for privacy in GC and testing is governed by the EU General Data Protection Regulation (EU Regulation 2016/679, GDPR) and the 2019 decision issued by the Italian Data Protection Authority (IDPA). These regulations stipulate that genetic data may be processed to protect the health of a blood relative, as long as the individual consented to such processing. However, the IDPA Decision remains silent on the possibility of the unsolicited disclosure of such information to at-risk relatives.

As a result, genetic data in Italy is considered the exclusive property of the carrier, and healthcare professionals are not legally authorized to share this information without the patient’s consent. While this approach safeguards individual privacy, it often leads to uncommunicated risks within families and missed opportunities for preventive interventions [39].

These critical issues may ultimately frustrate the efforts of healthcare professionals and undermine the ultimate purpose of genetic counselling; that is, to prevent the development of cancer in healthy but genetically predisposed individuals by tailoring diagnostic and interventional techniques to high-risk conditions.

A single Italian study [40] examined whether women who had undergone genetic testing intended to share their oncogenic risk with their relatives. The findings revealed a correlation between the intention to share genetic information and clarity of the genetic information received. Additionally, this study highlights the influence of family dynamics, including relationship and communication styles, as well as family structure, on the willingness to disclose such information. Supporting these findings, a recent systematic review and meta-analysis demonstrated that oncogenetic risk information is shared with family members by the proband in only 35% of cases (24–48%, 95% CI) [10]. This percentage is even lower in Italy, declining to a little over 20% [11].

Building on this evidence, the objective of this study was to gain insight into the challenges experienced by BRCA1/2 carriers when conveying genetic information within their family contexts. This investigation explored the variations in these challenges by considering the perspectives of probands/relatives, cancer patients/healthy subjects, and TGC/MCG subjects.

## Methods

### Aims

The present study aimed to examine the relationship between communication difficulties perceived by carriers and three key factors: the presence/absence of oncological disease, understanding of the genetic information received and type of oncological genetic counselling pathway accessed by carriers (TGC vs. MCG). Communication was explored in a large Italian sample of BRCA1/2 carriers, focusing on two factors that have been identified as influential: perceived emotional distress and understanding of genetic information.

### Design and setting of the study

The present study employed a prospective, observational, non-interventional design and its protocol was approved by the Ethics Committee of IRCCS Istituto Tumori Giovanni Paolo II of Bari (Prot.134/CE). IRCCS Istituto Tumori Giovanni Paolo II of Bari, which is the principal research institution, oversees the coordination of the various research activities conducted by the IRCCS Azienda Ospedaliero-Universitaria di Bologna (BO), IRCCS Ospedale Sacro Cuore Don Calabria di Negrar di Valpolicella (VR), Università Cattolica del Sacro Cuore and Fondazione Policlinico Universitario Agostino Gemelli, IRCCS (Roma). Thus, the study data were gathered in collaboration with aBRCAdaBRA ETS.

### Characteristics of participants and description of materials

To be eligible for inclusion, participants had to satisfy the following criteria: male and female individuals aged between 18 and 80 years, who tested positive for a BRCA1/2 PV and the capacity to understand the Italian language. Participant enrolment occurred from July 2022 to October 2023. These participants were affiliated with all mentioned institutions and the inclusion criteria demanded that participants have received their genetic test disclosure a minimum of one month prior to the trial start date. All patients provided a signed a IRB-approved informed consent. Those who did not meet the eligibility criteria were excluded from participation.

The study cohort comprised individuals carrying BRCA1/2 PVs, regardless of whether they were:

1. Probands, that is cancer patients or healthy individuals who had complete BRCA1 and BRCA2 testing and were the first in their family in whom the presence of a BRCA1/2 PV was identified;

## Relatives, i.e. healthy individuals or cancer patients who underwent cascade genetic testing due to the previous identification of a BRCA1/2 PV in their family.

After providing informed consent, participants completed a socio-demographic form, a self-administered survey and two evaluation questionnaires.

The sociodemographic form investigated the following characteristics: age, sex, marital status, educational background, occupation, BRCA1/2 status, proband/relative status, current state of health, any previous cancer diagnosis and, if applicable, tumor site, and inclusion in a GC or MCG pathway.

Moreover, the administered questionnaires included:

-Impact of Event Scale – Revised (IES-R) questionnaire [41]. The revised version of the most widely used a psychodiagnostic instrument is designed to assess the psychological impact of traumatic events. It is a 22-item scale rated on a 0 (not at all) to 4 (extremely) scale with respect to how distressing each item has been during the past week. The IES-R global scores were influenced by the subjects'responses to the three subscales of Intrusion (eight items), Avoidance (eight items) and Hyperarousal (six items). These subscales reflect the primary symptoms of post-traumatic stress disorder (PTSD) and demonstrate a high degree of intercorrelation [42]. Scores obtained on the Global IES scale were used to identify the degree of psychological distress experienced by an individual. The scale categorises scores into four distinct levels: normal (0–23), mild (24–32), moderate (33–36), and severe (> 37) [42]. It has been determined that scores above 50 likely indicate a state of probable PTSD [43].

The IES-R has been translated and validated in Italian, and its psychometric properties have been demonstrated to be satisfactory.

-The Distress Thermometer (DT) [44] is a single-item anchored instrument that assesses the subjective distress perceived by the patient through a visual analog scale (0 to 10 rank points).

The survey was developed ad hoc by the research team in order to compensate for the lack of validated instruments to investigate the variables explored in this study. The survey was constructed according to good qualitative research practice in psychology through a focus group process, that involved oncologists, geneticists, psycho-oncologists, general practitioners and patients. This survey was completed by both probands (Suppl.1) and relatives (Suppl.2) and investigated the understanding of genetic information, its dissemination to family members, modes and timing of transmission, communication issues and associated emotions or fears. In particular, the comprehension of genetic information obtained from the counselling was investigated by incorporating selected items from the Multidimensional Impact of Cancer Risk Assessment (MICRA), the first validated measure identified to capture this data [45, 46]. The incorporated items were explored using a five-points Likert scale for the comprehension of:

1.genetic information received.

2.one self cancer risk.

3.one’s relatives cancer risk.

4.one self’ risk management and reduction strategies.

The participants’ scores obtained in response to the comprehension items were assessed both separately and as a whole (global comprehension score on a five points Likert scale).

The probands were also asked whether they had encountered difficulties in conveying genetic information to their at-risk relatives. If so, they were invited to describe the nature of these difficulties. They were permitted to indicate one or more of the following options:"I find it difficult to explain complex health issues to others";"I lost contact with some/all of my family members";"I have a poor relationship with some/all of my family members";"I felt responsible or guilty";"I was afraid of their reactions/emotions"; and"Other."Additionally, probands were also asked whether, after receiving the genetic diagnosis, they became aware that the PV in question was already known within the family. Those who were unaware of this information prior to the diagnosis were classified as"unaware probands."

Processes, interventions and comparisons.

To facilitate the administration of the questionnaires in the various centers involved, each center was permitted to submit them to the participants in either paper form or via the Google Form that had been prepared for this purpose. Data from the administration of the questionnaires were collected and stored in a dedicated database.

## Statistical analysis

For the comparison of categorical variables, Pearson’s Chi-squared test or Fisher’s exact test was used, depending on the sample size and the distribution of expected frequencies. The Fisher’s exact test was used for low-frequency events. In this case, the number and frequencies are reported to summarize the data.

For continuous variables, the Wilcoxon rank-sum test was used, because the data did not follow a normal distribution. This non-parametric test was chosen to compare groups without assuming normality, making it appropriate for skewed or non-normally distributed data. In this case, the median and interquartile range (IQR) are reported to summarize the data.

Statistical analysis was performed through R v.4.4.1 environment, using the gtsummary package (v.2.0.2) [47].

## Results

The present study enrolled 277 BRCA1/2 carriers (115 probands and 162 relatives) who underwent TGC in 79.4% of cases and MCG in the remaining 20.6%. The characteristics of the sample are described in Table 1, including age, sex, health status, BRCA1/2 status and the type of genetic counselling pathway performed. Relatives resulted significantly younger than probands and, while the gender distribution was comparable in the two subgroups (probands vs. relatives), a significant difference was observed in the distribution of health status. Specifically, cancer patients were significantly more numerous among probands (83%) than among relatives (22%).

**Table 1:** Sample characteristics description

	Overall sample N = 277		p-value^1^
Characteristic	Relative, N = 162 (58.5)	Probands, N = 115 (41.5)	
Age	44 (35,50)^2^	47 (41,56)^2^	0.002^3^
Sex			0.062^1^
F	145 (90)	110 (96)	
M	17 (10)	5 (4.3)	
Health status			< 0.001^1^
Cancer patient	36 (22)	96 (83)	
Healthy subject	126 (78)	19 (17)	
Genetic Counselling			0.3^1^
MCG	30 (19)	27 (23)	
TGC	132 (81)	88 (77)	
BRCA status			0.9^1^
BRCA1	83 (51)	62 (54)	
BRCA2	75 (46)	51 (44)	
BRCA1 and BRCA2	4 (2.5)	2 (1.7)	

^1^Pearson's Chi-squared test; Fisher's exact test—^2^ n (%)

^2^ Median (Q1, Q3)

^3^Wilcoxon rank sum test

IES-R scorings obtained by participants are described in Table 2 and Table 3.

**Table 2:** IES-R scorings (global score, IES-Avoidance, IES-Hyperarousal, IES-Intrusion) in proband and relatives

	Relatives, N = 162	Probands, N = 115	p-value^2^
IES Avoidance	8.0 (4.0, 12.8)	8.0 (4.0, 13.0)^1^	0.8
IES Hyperarousal	9.0 (4.0, 14.0)	11.0 (6.0, 16.0)	0.030
IES Intrusion	8.0 (3.0, 13.0)	11.0 (5.0, 16.5)	0.039
IES global score	25.5 (11.0, 39.0)	32.0 (17.0, 43.0)	0.064

^[1]^Median (IQR)—^2^ Wilcoxon rank sum test

**Table 3:** IES-R scores of probands and relatives described in consideration of their health status, specifically distinguishing between cancer patients and healthy individuals

	Probands			Relatives		
	Cancer patient, N = 96	Healthy subjects, N = 19	p-value^1^	Cancer patient, N = 36	Healthy subjects, N = 126	p-value^1^
IES- A	8.00 (4.00, 13.00)^2^	6.00 (3.50, 9.00)	0.3	10.50 (5.75, 14.00)	7.00 (4.00, 12.00)	0.042
IES- H	12.00 (6.00,16.00)	7.00 (4.50, 14.50)	0.10	13.00 (5.75, 17.25)	8.00 (4.00,13.75)	0.028
IES- I	11.00 (5.00, 17.00)	9.00 (0.50, 12.00)	0.066	12.00 (7.00, 20.00)	7.00 (2.00, 12.00)	0.001
IES- global	32.00 (18.00,43.25)	21.00 (10.50,36.00)	0.069	34.50 (22.25, 50.25)	23.50 (10.00,37.00)	0.008
IES PTSD						
< 33	49 (51%)	13 (68%)	0.2	18 (50%)	86 (68%)	0.044
≥ 33	47 (49%)	6 (32%)		18 (50%)	40 (32%)	

^1^Pearson's Chi-squared test; Fisher's exact test; Wilcoxon rank sum test – ^2^Median (IQR)

A significant difference was observed in the IES subscale scores between probands and relatives on the Hyperarousal (p-value IES-H 0.030) and Intrusiveness (p-value IES-I 0.039) subscales, as illustrated in Table 2.

Moreover, when the cohort was examined in relation to the state of health of its members (Table 3) it was evident that the affected relatives exhibited considerably higher global scores (p-value 0.008), intrusiveness (p-value IES-I 0.001), avoidance (p-value IES-A 0.042), and hyperarousal (p-value IES-H 0.028) scores than healthy relatives. Similarly, a statistical trend was identified in probands affected by cancer in comparison to healthy individuals (Table 3), with regard to total scores (p = 0.069) and intrusiveness (IES-I, p = 0.066). Regardinf the clinical psychological significance of the global IES-R scores, it should be noted that no cases of severe psychological distress or PTSD were diagnosed in either of the groups. However, in our cohort the likelihood of psychological distress reactions was greater in probands than in relatives, and in both groups, the presence of these reactions appeared to be more linked to an underlying oncological condition.

Furthermore, Table 4 shows that the distress thermometer (DT) scores observed in probands were markedly higher than those recorded in relatives (p-value 0.045). Conversely, no notable statistical discrepancy was identified in the perceived distress levels of the participants who had undergone TGC and MCG.

**Table 4:** Description of Distress Thermometre (DT) scorings in the cohort

	Relatives, N = 162^1^	Probands, N = 155^1^	p-value^2^	TGC	MCG	p-value^2^
Distress Thermometre (DT)	6.0 (4.0, 8.0)^3^	7.0 (5.0, 8.0)	0.045	6.00 (4.00, 8.00)	7.00 (4.00, 8.00)	0.8

^1^Number—^2^ Wilcoxon rank sum test – ^3^ median (IQR)

Analysis of the responses to the survey question"Do you clearly understand the genetic information you received?"indicates that the level of comprehension of the genetic information received (Table 5) was significantly higher in subjects who underwent TGC than in those who received MCG (p < 0.001 Fisher extract test; p < 0.001 Wilcoxon rank sum test).

**Table 5:** Description of the whole sample distribution of responses to the question"*Do you clearly understand the genetic information you received*?"among individuals who have undergone either traditional genetic counseling (GC) or mainstream cancer genetics (MCG)

	MCG, N = 57	TGC, N = 220	p-value
Comprehension			< 0.001^2^
0	2 (3.5%)^1^	0 (0%)	
1	2 (3.5%)	2 (0.9%)	
2	5 (8.8%)	1 (0.5%)	
3	3 (5.3%)	6 (2.7%)	
4	12 (21%)	25 (11%)	
5	33 (58%)	186 (85%)	
	4.11 (1.37)	4.78 (0.60)	< 0.001^3^

^1^n (%);^2^Fisher's exact test; ^3^Wilcoxon rank sum test

Moreover, an analysis of the responses provided by the two groups of participants to the same question (Table 6) indicates a significantly enhanced comprehension of genetic information among relatives compared to probands (p = 0.007).

**Table 6:** Description of the differences in understanding between the genetic information of probands vs relatives, as outlined in response to the query"*Do you clearly understand the genetic information you received*?"

	Relative, N = 162 (58.5)^1^	Proband, N = 115 (41.5)	p-value^2^
Comprehension of genetic information	4.74 [median: 5.0] (5.0, 5.0)^3^	4.50 [median: 5.0] (4.0, 5.0)	0.007

[1] n (%)—^2^Wilcoxon rank sum test- ^3^average [median] (IQR)

A comparative analysis of the responses given by the probands to all survey items pertaining to their comprehension of genetic information received in TGC vs MCG (Table 7) revealed that those who underwent TGC had a more comprehensive understanding of the genetic data received (p = < 0.001), personal cancer risk (p = 0.006), and existing risk management strategies (p = 0.001) than those who underwent MCG.

**Table 7:** The distribution of probands'responses to questions regarding their comprehension of genetic information received during GC or MCG

Probands’ responses	MCG, N = 27	TGC, N = 88	p-value^1^
Understanding genetic information			< 0.001
0	1 (3.7%)^2^	0 (0%)	
1	2 (7.4%)	0 (0%)	
2	3 (11%)	1 (1.1%)	
3	3 (11%)	3 (3.4%)	
4	7 (26%)	13 (15%)	
5	11 (41%)	71 (81%)	
Personal cancer risk			0.006
1	0 (0%)	1 (1.1%)	
2	3 (11%)	0 (0%)	
3	1 (3.7%)	1 (1.1%)	
4	7 (26%)	13 (15%)	
5	16 (59%)	73 (83%)	
Relatives’ cancer risk			0.066
0	0 (0%)	1 (1.1%)	
1	1 (3.7%)	1 (1.1%)	
3	4 (15%)	5 (5.7%)	
4	7 (26%)	11 (13%)	
5	15 (56%)	70 (80%)	
Risk management strategies			0.001
1	1 (3.7%)	1 (1.1%)	
2	3 (11%)	0 (0%)	
3	2 (7.4%)	2 (2.3%)	
4	7 (26%)	12 (14%)	
5	14 (52%)	73 (83%)	
Global comprehension scores	17.00 (15.00, 20.00)	20.00 (18.00, 20.00)	< 0.001

1 Fisher's exact test; Wilcoxon rank sum test; ^2^ n (%);

Similarly, analysis of relatives'comprehension of the genetic information received (Table 8) demonstrates that relatives who underwent TGC showed a more comprehensive understanding of genetic data (p = 0.018), personal cancer risk (p = 0.049), and available risk management strategies (p = 0.022) than those who underwent MCG.

**Table 8:** The distribution of relatives’ responses to questions regarding their comprehension of the genetic information received during GC or MCG

Relatives’ responses	MCG, N = 30	TGC, N = 132	p-value^1^
Understanding genetic information			0.018
0	1 (3.3%)	0 (0%)	
1	0 (0%)	2 (1.5%)	
2	2 (6.7%)	0 (0%)	
3	0 (0%)	3 (2.3%)	
4	5 (17%)	12 (9.1%)	
5	22 (73%)	115 (87%)	
Personal cancer risk			0.049
0	1 (3.3%)	0 (0%)	
1	1 (3.3%)	2 (1.5%)	
2	1 (3.3%)	0 (0%)	
3	2 (6.7%)	3 (2.3%)	
4	3 (10%)	14 (11%)	
5	22 (73%)	113 (86%)	
Relatives’ cancer risk			0.082
1	0 (0%)	3 (2.3%)	
2	2 (6.7%)	1 (0.8%)	
3	3 (10%)	4 (3.0%)	
4	3 (10%)	13 (9.8%)	
5	22 (73%)	111 (84%)	
Risk management strategies			0.022
1	0 (0%)	4 (3.0%)	
2	1 (3.3%)	1 (0.8%)	
3	6 (20%)	5 (3.8%)	
4	4 (13%)	22 (17%)	
5	19 (63%)	100 (76%)	
Global comprehension scores	20.00 (17.25, 20.00)	20.00 (19.00, 20.00)	0.054

^1^ Fisher's exact test; Wilcoxon rank sum test; ^2^ n (%);

Finally, the results of the survey demonstrated that BRCA1/2 carriers frequently encountered communication difficulties (Table 9).

**Table 9:** Distribution of unaware probands in our sample

	Unaware Probands	
After you received your genetic diagnosis, you discovered that other relatives already knew about the BRCA1/2 pathogenetic variant in your family?	No, N = 97^1^ (84%)	Yes , N = 18^1^ (16%)

Specifically, 18 of the 115 probands within the sample reported that their BRCA1/2 PV was already known within their family before they discovered it (16% of unaware probands). This deficiency in disclosure of genetic test results and cascade genetic testing prevented 14 out of 18 (77.8%) unaware probands from reducing their cancer risk. Consequently, these subjects were diagnosed with cancer.

## Discussion

Disclosure of genetic test results in BRCA1/2 carriers is crucial for of cascade genetic counselling and testing [48, 48–51]. International literature shows that it is influenced by many factors [51–53]. Among these factors, both the emotional distress experienced by patients [48, 51] and the level of understanding of the genetic principles of risk transmission among probands [50, 52] play key roles. To the best of our knowledge, this is the first study to investigate these two variables using a large sample of Italian BRCA1/2 carriers.

First, the findings demonstrate that mild and moderate post-traumatic stress symptoms (hyperarousal and intrusiveness IES-subscales) and perceived distress (DT scorings) were significantly more prevalent in probands than in relatives. The proposed underlying hypothesis is that, in contrast to relatives, probands are required to concurrently cope with multiple stressful events. In fact, in probands, cancer diagnosis often coincides with the diagnosis of genetic hereditary cancer syndrome: in our sample 96 out of 115 probands (83%) had cancer. Consequently, probands must concurrently cope with cancer, understand their own possibilities managing their personal high risk of cancer and cope with the emotions connected with the moral responsibility of informing their at-risk relatives. The psychological burden [25, 27–29] that probands experience may, therefore, exceed their resilience capacities resulting in mild physical (hyperarousal), cognitive (intrusiveness) and behavioural (avoidance) post-traumatic stress symptoms, as well as the perception of personal distress, may be more likely.

Furthermore, our findings suggest that mild to moderate post-traumatic stress symptoms manifest more frequently in cancer patients than in healthy individuals. This observation was consistent across both the relative and proband subgroups. Consequently, it can be hypothesised that this outcome may be attributable to the development of the malignancy itself rather than being a consequence of the genetic diagnosis.

These results, together with the hypotheses formulated, are consistent with the results of our previous work [54] as well as with those of the international literature [25, 55, 56] on the psychological burden experienced by BRCA1/2 carriers who have been diagnosed with cancer, compared to healthy ones.

A comprehensive analysis of the participants'responses also revealed a marked discrepancy in the comprehension of genetic information, with relatives demonstrating a significantly more profound understanding of the subject than probands. This finding can be interpreted in the light of the hypothesis already formulated regarding the cognitive and emotional overload experienced by probands who undergo genetic counselling in the same context as their cancer diagnosis. Indeed, relatives were more frequently found to be cancer-free than probands (78% and 17% respectively). Furthermore, they experience a reduced degree of emotional distress when it comes to the moral responsibility of passing genetic information. As demonstrated by existing literature research [57, 58], the cognitive load for patients acquiring and processing new complex health-related information is high and emotions have a considerable impact on challenging cognitive processing. In fact emotional distress has a significant influence on attention and learning processes based on comprehension [59], and acute stress is associated with an increased incidence of mind wandering and concomitant deterioration in cognitive performance [60]. Moreover, the degree of emotional distress experienced may have a detrimental effect on attention and concentration test performance, thereby altering patients'focus [61]. Therefore, it can be hypothesized that proband who are already burdened by the emotional impact of cancer-related distress and the volume of complex health and genetic information received may experience greater challenges in attention and comprehension when receiving genetic diagnosis.

The most intriguing outcome of the present study pertains to the disparities in comprehension observed among BRCA1/2 carriers who underwent TGC compared to those who did not. In fact, TGC probands and relatives demonstrated a more comprehensive understanding of the genetic data received, their personal cancer risk, and available risk management strategies.

In this renard, two clarifications are required. First, the administered survey had to be clear and easily comprehensible to the participants. Therefore, to distinguish subjects who had undergone TGC or MCG, the experimenters invited them to answer the following question: “Have you undergone a genetic counselling consultation with a geneticist?”. Participants were invited to select one of the following options:

“Yes, I have undergone cancer genetic counselling with a geneticist.”;

“No, I performed the genetic test directly on the instruction of a specialist doctor.”.

Considering the participants'reports, an investigation was conducted to ascertain whether the disclosure of genetic test results occurred subsequent to the TGC or in the aftermath of genetic testing alone (as evident from the Mainstream Cancer Genetics procedure).

Furthermore, the Medical Genetics centers participating in the study only enrolled subjects (both probands and relatives) who had undergone TGC at their health service, whereas the National Association aBRCAdaBRA ETS had the possibility of enrolling also BRCA carriers who had only undergone a genetic test. Althought the involvement of the aBRCAdaBRa association enabled the recruitment of a larger and more diverse sample of participants, this approach concomitantly reduced researchers’ accessibility to information pertaining to the healthcare procedures undertaken by study participants. Therefore, the underlying assumption of our analysis was that subjects who were referred to have undergone genetic testing alone could be included in what has come to be known over time as MCG in order to distinguish them from those who had undergone TGC.

In light of this assumption, it is conceivable that this result is related to the nature of the genetic counselling process that the participants underwent (TGC vs. MCG). TGC typically involve a longer period of time and at least two consultations with a geneticist or genetic counsellor who builds a supportive relationship with the suspected PV carrier and investigates their personal and family history for prevention purposes [16, 17]. In contrast, the MCG model is driven by the need for oncologists/gynaecologists/surgeons/other specialists to quickly refer a patient for genetic testing for therapeutic decisions after a session that is usually shorter than the TGC [18, 19]. From the patient’s perspective, this paradigm shift has reduced opportunities to receive tailored information about the likelihood of being a carrier, implications for extended family members, and the process of making a gradual informed decision regarding genetic testing [62]. As highlighted by recent research, women undergoing BRCA1/2 genetic testing through the MCG model had lower knowledge scores than those following the standard genetic counselling pathway [62]. It has also been reported that the mainstreaming approach does not allow sufficient time to make an informed decision about genetic testing [63]. Indeed, the prior finding regarding the heightened comprehension among relatives compared with probands can be attributed to the distinctions between TGC and MCG. Relatives are significantly more likely to be referred to a geneticist in the TGC owing to the heightened risk of cancer. Conversely, it is more plausible that probands with cancer seek MCG for therapeutic interventions.

In addition, the study results showed a lack of significant difference in the perceived understanding of relatives'cancer risk among probands who underwent TGC versus MCG. This finding could be related to the degree of uncertainty and both emotional and cognitive overload experienced by probands in relation to the disclosure of their genetic outcomes. As discussed above, cognitive abilities may be less efficient when subjects are simultaneously confronted with both complex health data and large amounts of information. Indeed, it may be hypothesized that upon disclosure of genetic test results, the primary focus for probands may be their own cancer risk, as opposed to that of their relatives (whose perception of understanding is equally impaired irrespective of the communicative competence of healthcare providers and the timing of counselling).

It is also important to note that given the specific aspects of cancer genetic counselling, the National Human Genetics and Medical Oncology Scientific Societies (SIGU and AIOM) recommend that Italian centres offering TGC employ professionals with integrated expertise, including geneticists, specialists in preventive medicine, cancer diagnosis and treatment, and psychologists [64]. The presence of several specialists is a minimum requirement for the provision of TGC, as opposed to the MCG model. We hypothesise that this enables different professional skills to be used to improve the effectiveness of physician–patient communication. In contrast, non-genetic health professionals involved in the MCG model are not always familiar or confident with genetic counselling, often report difficulties in managing patients'emotions [65, 66], and are willing to receive specific training to improve their communication skills [67].

A notable finding of the present study is the number of relatives who, in contrast to the predictions, did not undergo TGC (30/162, 18,5%). This finding is particularly remarkable because only a small proportion of these subjects were cancer patients (5/30, 16.6%), however they reported receiving the genetic test alone rather than the TGC. It is possible to hypothesize that this surprising result may be related to the limited availability of medical genetic services in the country. Indeed, given that family members who did not undergo TGC were exclusively enrolled through the aBRCAdaBRA association and are thus part of a context of high information and awareness, it is plausible to argue that, even if they were prevented from accessing medical genetics, they were nevertheless able to access genetic testing alone, even in the absence of the disease, because their BRCA family had already been addressed from an oncological point of view.

The finding that a significant proportion of the probands in our sample were unaware of their BRCA1/2 status (16%) provides further evidence of the probands'need for targeted informational and emotional support in receiving, understanding and disseminating genetic information to their at-risk family members. In fact, 14 of the 18 (77.8%) unaware probands (participants who, after receiving their genetic diagnosis, discovered that the hereditary cancer risk syndrome was already known in their family) had already developed a malignancy at the time of testing. This is a particularly relevant issue, as it represents a failure of the fundamental purpose of oncological genetic counselling to empower individuals at elevated hereditary cancer risk to implement clinical and instrumental prevention strategies. Consistent with scientific research, this result shows the inadequacy of cascade genetic testing and counselling rates [68]. It also demonstrates the importance of implementing intrafamilial dissemination of genetic information [48–50] through psychosocial support interventions that promote both probands and health professionals communication skills [68, 69].

## Conclusions

The present study investigated the intra-familial transmission of genetic diagnosis in a large Italian sample of BRCA1/2 carriers, focusing on two factors that have been identified as influential: perceived emotional distress and understanding of genetic information.

First, our results provide further evidence, within the Italian context, of the findings from international research on the psychological impact of genetic diagnosis and the distress experienced by BRCA1/2 carriers, thereby underlining the pivotal role of cancer in triggering mild-to-moderate post-traumatic symptoms and distress-related manifestations. Furthermore, our findings unequivocally demonstrate that individuals carrying a BRCA1/2 PV who undergo TGC acquire a more comprehensive and detailed comprehension of the genetic information received compared to MCG. While MCG is undoubtedly valuable and essential in addressing the rising demand for early access to genetic testing for therapeutic purposes, our findings emphasize the necessity of referring all BRCA1/2 carriers to genetics specialists to fully realize the cancer prevention objective of genetic counselling itself. As we look into the future, we need to critically approach MCG, determine how to address carriers understanding and prevention needs and reincorporate a more comprehensive genetic risk assessment into the MCG model.

The value of the present study lies in its findings being consistent with previous literature, yet offering a unique perspective on the personal experience of BRCA1/2 carriers who underwent cancer genetic counselling. Indeed, previous studies that have examined the dynamics associated with cascade genetic counselling and testing focusing on the experiences of healthcare professionals have identified the need to enhance their communication and interpersonal skills. In this respect, our study offers a novel perspective on the scientific research landscape by underscoring the necessity of personalized support for BRCA1/2 carriers when it comes to comprehending and disseminating genetic information. Consequently, it is essential to provide customized interventions that offer emotional and communication support to healthcare professionals involved in genetic counselling, as well as to their patients. This is particularly crucial considering the suboptimal rates of cascade genetic counselling and testing observed among BRCA1/2 carriers. Thus, in contrast to previous studies that predominantly focused on the uptake rate of cascade counselling and testing, the present study focused on the self-reported experiences of Italian BRCA carriers. Given the substantial volume of data collected, this study was specifically concerned with the perceived understanding of information by both probands and their relatives. A qualitative analysis of the various challenges reported by BRCA carriers is postponed for subsequent studies that are currently being drafted.

The limitations of the present study can be attributed to two main factors. First, the relatively small sample size, and secondly, the absence of a validated questionnaire for measuring the true understanding of the genetic information received by BRCA carriers. The survey administered in this study sought to assess the extent to which BRCA carriers believed that they had comprehended the implications of their genetic diagnosis. Nevertheless, a discrepancy may emerge between the carriers'perceived comprehension and the correctness of their genetic information. Therefore, it would be desirable to replicate the results emerging from the present study using larger sample and also to measure the truthfulness and accuracy of the information held by BRCA carriers.

Further studies should investigate how to improve the communication of genetic information to at-risk relatives by BRCA1/2 proband, while also addressing the need to better understand additional personality and family psychological variables that may impact the understanding of genetic risk by BRCA1/2 carriers.

## Fundings

Progetto 5 × 1000 – Ministero della Salute – dal titolo Ricerca di potenziali marcatoripredittivo/prognostici tissutali e circolanti in pazienti con adenocarcinoma del pancreas e delle vie biliari intra ed extraepatiche nei setting adiuvante e metastatico – CUP: F93 C23000120001 – delib n.303/2023 – P.I. Oronzo Brunetti.

## Supplementary Information

Supplementary Material 1.

Supplementary Material 2.

## Data Availability

The datasets used and/or analysed during the current study are available from the corresponding author on reasonable request.
