# Supplementary material for: BRCA cascade counselling and testing in Italy: current position and future directions
Source: BMC Cancer. 2025 Jul 1;25:1044. doi: 10.1186/s12885-025-14419-y (PMC12210843; doi:10.1186/s12885-025-14419-y)
Supplement: Supplementary file 1 — Supplementary Material 1. [file 12885_2025_14419_MOESM1_ESM.docx]

**Suppl.1 Probands survey**

**1. After you received your genetic diagnosis, you discovered that other relatives already knew about the BRCA1/2 pathogenetic variant in your family? (unaware proband)**

□ yes □ no

**2.When did you receive your genetic diagnosis?**

□ 1-3 months ago

□ 4-6 months ago

□ 7-12 months ago

□ more than 12 months ago

**3.** **Were you included in a traditional genetic counselling process with a geneticist or genetic counsellor?**

□ yes, I had a oncological genetic counselling with a geneticist/genetic counsellor

□ No, I directly performed the genetic test on a specialist doctor's prescription

**4.** **Do you clearly understand the genetic information you received??**

Please indicate your degree of genetical information understanding with a number from 1 (I did not understand at all) to 5 (I fully understood)

□0 □1 □2 □3 □4 □5

**5.** **Do you clearly understand your current risk of developing cancer?**

Please indicate your degree of genetical information understanding with a number from 1 (I did not understand at all) to 5 (I fully understood)

□0 □1 □2 □3 □4 □5

**6.Do you have a clear understanding of your relatives' risk of developing cancer?**

Please indicate your degree of genetical information understanding with a number from 1 (I did not understand at all) to 5 (I fully understood)

□0 □1 □2 □3 □4 □5

**7. Have you received instructions on the most appropriate strategies for managing/reducing your cancer risk??**

□ yes

□ no

**8.Have you clearly understood which strategies are most appropriate to manage or reduce your cancer risk?)**

Please indicate your degree of genetical information understanding with a number from 1 (I did not understand at all) to 5 (I fully understood)

□0 □1 □2 □3 □4 □5

**9.Have you received instructions on which family members to inform of the presence of a genetic mutation in the family?**

□ yes

□ no

**10.Have you informed your family members of the presence of a genetic mutation in your family?**

□ no

□ yes, all of them

□ yes, some of them

**10.0 Why ?________________________________________________________________________**

**10.1 If yes, did you choose a particular time to inform your family members?**

□ no, I informed the mas soon as I could

□ yes, I waited for the right moment

If yes, when?_______________________________________________

**10.2 How long after the genetic report did you inform your family??**

□ 1-3 months

□ 3-6 months

□ 6-12 months

□ > 12 months

**10.3 In che modo ha informato i suoi familiari?**

□ li ho incontrati di persona

□ li ho contattati telefonicamente

□ li ho contattati via mail

**11. Did you experience any challenges in passing on the genetic information to your at-risk relatives?**

□ no, at all

□ yes, a few difficulties

□ yes, many difficulties

**11.1 If yes, which challenges you expeienced?**

□ I find it difficult to explain complex health issues to others

□ I lost contact with some/all of my family members

□ I have bad relationship with some/all of my family members

□ I felt responsible or guilty

□ I was afraid of their reactions/emotions

□ other ____________________________________________________________

**12. How have you chosen to manage your cancer risk? (you can tick more than 1 box)**

□ I undertake intensive breast checks (every 6 months for women, annually for men)

□ I undertake intensive gynaecological examinations (every 6 months)

□ I do pharmacological prevention

□ I undertake annual urological check-ups

□ I undertake annual dermatological check-ups

□ I perform dosage of tumour markers

□ I underwent prophylactic breast surgery

□ I underwent prophylactic gynaecological surgery

□ other:________________________________________________________

**13. What is your level of stress in relation to the genetic diagnosis you received?**

Please indicate your current degree of stress in relation to the genetic information received with a number from 0 (I am not stressed at all) to 10 (I am very stressed indeed)

□0 □1 □2 □3 □4 □5 □6 □7 □8 □9 □10

***14. If you find it useful, you can leave your name and telephone number to be re-contacted by one of the psycho-oncologists in charge of this study and arrange a telephone or video call.***

**MAKE SURE YOU HAVE ANSWERED EVERY QUESTION. THANK YOU.**
