# Supplementary material for: BRCA cascade counselling and testing in Italy: current position and future directions
Source: BMC Cancer. 2025 Jul 1;25:1044. doi: 10.1186/s12885-025-14419-y (PMC12210843; doi:10.1186/s12885-025-14419-y)
Supplement: Supplementary file 2 — Supplementary Material 2. [file 12885_2025_14419_MOESM2_ESM.docx]

**Suppl.2 Relatives survey**

**1.** **When did you learn that there was a genetic mutation in your family?**

□ 1-3 month ago

□ 4-6 month ago

□ 7-12 month ago

□ more thani 12 month ago

**2.** **Who informed you?**

□mother □father □sister □brother □aunt □uncle □cousin(female) □cousin(male) □other___________________________

**3.** **How long did it take between the moment your family member received a genetic diagnosis and the moment you became aware of it?**

□ 1-3 months

□ 3-6 months

□ 6-12 months

□ > 12 months

**4. Were you informed of your family's genetic diagnosis in a particular moment?**

□ no, they informed me as soon as they could

□ yes, they waited for the right moment

**4.1 if yes, which moment?_______________________________________________**

**5. How did your relatives inform you?**

□ I was told about it in person

□ I was phone-called

□ I was e-mailed

**6.** **Were you included in a traditional genetic counselling process with a geneticist or genetic counsellor?**

□ yes, I had a oncological genetic counselling with a geneticist/genetic counsellor

□ No, I directly performed the genetic test on a specialist doctor's prescription

**7.** **Do you clearly understand the genetic information you received??**

Please indicate your degree of genetical information understanding with a number from 1 (I did not understand at all) to 5 (I fully understood)

□0 □1 □2 □3 □4 □5

**8.** **Do you clearly understand your current risk of developing cancer?**

Please indicate your degree of genetical information understanding with a number from 1 (I did not understand at all) to 5 (I fully understood)

□0 □1 □2 □3 □4 □5

**9.Do you have a clear understanding of your relatives' risk of developing cancer?**

Please indicate your degree of genetical information understanding with a number from 1 (I did not understand at all) to 5 (I fully understood)

□0 □1 □2 □3 □4 □5

**10. Have you received instructions on the most appropriate strategies for managing/reducing your cancer risk??**

□ yes

□ no

**11.** **Have you clearly understood which strategies are most appropriate to manage or reduce your cancer risk?)**

Please indicate your degree of genetical information understanding with a number from 1 (I did not understand at all) to 5 (I fully understood)

□0 □1 □2 □3 □4 □5

**12. How did you feel receiving the genetic information from your family member? (you can tick more than one box)**

□ I felt anxious

□ I felt angry to my relative

□ I felt helpless

□ I felt afraid

□ I felt overwhelmed

□ I experienced it as an opportunity

□ other __________________________________

**12. How have you chosen to manage your cancer risk? (you can tick more than 1 box)**

□ I undertake intensive breast checks (every 6 months for women, annually for men)

□ I undertake intensive gynaecological examinations (every 6 months)

□ I do pharmacological prevention

□ I undertake annual urological check-ups

□ I undertake annual dermatological check-ups

□ I perform dosage of tumour markers

□ I underwent prophylactic breast surgery

□ I underwent prophylactic gynaecological surgery

□ other:________________________________________________________

**13. What is your level of stress in relation to the genetic diagnosis you received?**

Please indicate your current degree of stress in relation to the genetic information received with a number from 0 (I am not stressed at all) to 10 (I am very stressed indeed)

□0 □1 □2 □3 □4 □5 □6 □7 □8 □9 □10

***14. If you find it useful, you can leave your name and telephone number to be re-contacted by one of the psycho-oncologists in charge of this study and arrange a telephone or video call.***

**MAKE SURE YOU HAVE ANSWERED EVERY QUESTION. THANK YOU.**
